# Diagnostic Challenges in the Cytology of Thymic Epithelial Neoplasms

**DOI:** 10.3390/cancers14082013

**Published:** 2022-04-15

**Authors:** Jonathan Willner, Fang Zhou, Andre L. Moreira

**Affiliations:** Department of Pathology, NYU Langone Health, New York, NY 10016, USA; jonathan.willner@nyulangone.org (J.W.); fang.zhou@nyu.edu (F.Z.)

**Keywords:** Thymic cytology, thymus, cytology, fine needle aspiration, thymoma, thymic carcinoma, thymic epithelial neoplasm

## Abstract

**Simple Summary:**

Thymic epithelial neoplasms, including thymoma, thymic carcinoma, and thymic neuroendocrine neoplasms, constitute the majority of anterior mediastinal masses. Fine needle aspirations (FNA) of mediastinal masses are infrequently encountered and are highly challenging to interpret. Thymic neoplasms display a significant degree of histologic diversity and have overlapping morphologic features with tumors from other sites. However, when properly interpreted alongside ancillary studies and radiologic findings, FNAs can yield clinically actionable results. This review aims to illustrate the usefulness and diagnostic pitfalls of thymic FNAs to assist pathologists in analyzing these specimens.

**Abstract:**

Thymic epithelial neoplasms are rare tumors that constitute the majority of anterior mediastinal masses. They are classified as thymomas, thymic carcinomas, and thymic neuroendocrine neoplasms. Biopsy diagnosis is not common, and most tumors are surgically resected. Biopsy, including cytology, is indicated when a non-surgical entity is suspected or in cases of locally advanced disease. Smears of thymomas consist of round or spindle epithelial cells admixed with varying amounts of lymphocytes depending on the type of thymoma. Smears of thymic carcinoma and thymic neuroendocrine neoplasms are often indistinguishable from corresponding tumor types from other organs. Accurate cytological diagnosis can be difficult due to the histological diversity of thymomas, as well as the morphological features that certain thymic tumors share with similar tumors from other organs. However, fine needle aspiration (FNA) of anterior mediastinal masses can provide clinically actionable information and can be used to determine whether lesions require surgical, systemic, or local noninvasive treatments. Ancillary studies, namely, immunocytochemical stains, flow cytometry, and radiology, are important tools in the evaluation of thymic aspirates. This review discusses the utility and limitations of thymic FNAs and illustrates the diagnostic features and pitfalls of these specimens.

## 1. Introduction

Thymic epithelial tumors are classified as thymomas, thymic carcinomas, and thymic neuroendocrine neoplasms. Thymomas are subdivided based on epithelial cell morphology and lymphocyte-to-epithelial ratio. Thymic carcinomas and thymic neuroendocrine neoplasms morphologically resemble analogously named tumor types in other organs [1]. Lymphomas, metastatic carcinomas, and germ cell tumors can also affect the mediastinum [2]. Biopsy diagnosis is an important step in the evaluation of mediastinal masses. Early-stage primary thymic epithelial tumors may be managed by surgery. However, cases with local spread of disease or metastatic disease can be inoperable, and a biopsy confirmation of a thymic epithelial tumor may guide the decision on the best systemic therapy, including neoadjuvant therapy. Similarly, systemic and/or radiation therapy is usually the primary treatment modality for lymphomas, mediastinal germ cell tumors, and metastatic tumors [3,4,5].

Fine needle aspiration (FNA) is not commonly used in thymic tumors, despite being a minimally invasive diagnostic method that is generally well-tolerated. The reasons for this include: (1) the rarity of thymic neoplasms; (2) the challenges of FNA interpretation, particularly in non-specialized centers that have limited experience with these tumors; and (3) the possibility of tumor dissemination if the pleural space is traversed by the biopsy needle, although reports of needle track dissemination in the literature are rare [6,7,8,9].

Due to the rarity of thymic tumors, there is scant literature regarding the cytological features of even benign thymic lesions. However, thymic FNA has the potential to provide clinically actionable results by providing a diagnostic classification [10,11]. In this article, we review the utility, challenges, and differential diagnosis of cytopathology in the evaluation of thymic epithelial tumors (thymomas, thymic carcinomas, and neuroendocrine neoplasms) and we provide illustrative figures of important diagnostic points. 

## 2. Thymoma

Thymoma is a rare tumor with an estimated incidence of 0.13 per 100,000 persons per year [12], though it constitutes up to half of all anterior mediastinal masses [13]. Approximately half of patients with thymoma are asymptomatic, while the remainder present with paraneoplastic syndromes or mass effect. The most common paraneoplastic syndrome is myasthenia gravis, which is seen in up to 25–40% of thymoma cases [14,15]. Thymomas represent a group of tumors with heterogeneous histological patterns. The 2021 World Health Organization (WHO) classification of thoracic tumors categorizes thymomas as type A, type B with subcategories, and type AB (Table 1) [1].

**Figure 1:**
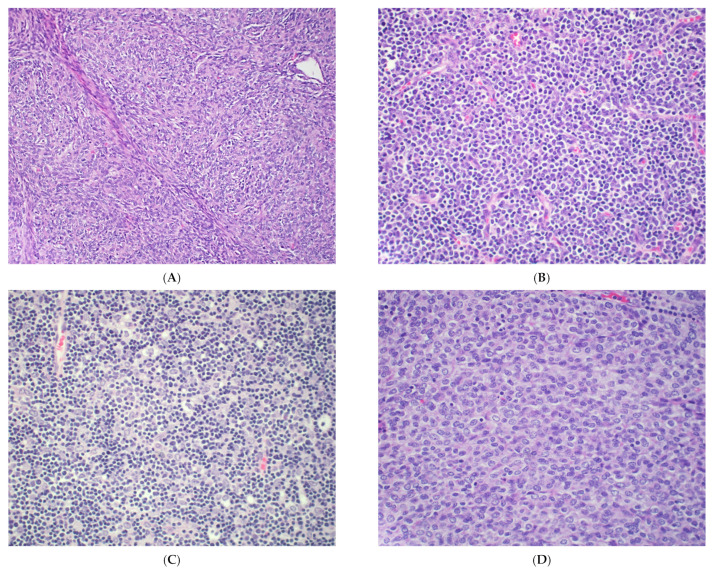
Histological subtypes of thymoma. (**A**) Type A thymoma with spindle epithelial cells. (**B**) Type B1 thymoma with rare epithelial cells and abundant lymphocytes. (**C**) Type B2 thymoma with epithelial islands admixed with lymphocytes. (**D**) Type B3 thymoma with round to ovoid epithelial cells and rare lymphocytes. All images show H&E stains, 20× objective.

Grossly, thymomas are circumscribed, often encapsulated, lobulated lesions with or without cystic degeneration. Capsular invasion and stage predict prognosis [16,17]. Histologically, type A thymoma is composed of spindled epithelial cells and rare lymphocytes, whereas type B thymoma shows variable proportions of polygonal epithelial cells and thymic lymphocytes (Figure 1). Type B thymoma is further divided into three subcategories according to the ratio of lymphocytes to thymic epithelial cells. Type B1 thymoma is rich in thymic lymphocytes and frequently contains medullary islands between the scattered epithelial cells. Type B2 thymoma is also lymphocyte-predominant and contains clustered epithelial cells. Medullary islands are generally absent, and a lobular growth pattern is observed. Type B3 is composed predominantly of epithelial cells with few lymphocytes. Type AB thymoma contains areas with both type A and type B characteristics, often with sharp transitions. Rarer types of thymoma include metaplastic thymoma, consisting of islands of epithelial cells separated by an abundant fibroepithelial spindle cell stroma, and micronodular thymoma with lymphoid hyperplasia, consisting of discrete nodular islands of ovoid to spindle epithelial cells separated by abundant lymphoid stroma with or without germinal centers [1].

Thymic epithelial cells express squamous markers (p40, p63, and cytokeratins) and markers of thymic origin (PAX8, FOXN1, and CD205) [1,18,19,20]. Thymic lymphocytes show reactivity for TdT, CD99, CD5, and CD3, which may be detected via flow cytometry [1]. CD5 is commonly expressed in the epithelial cells of thymic carcinomas, while in most thymomas, CD5 is seen exclusively in the lymphocytes [21]. These and other immunocytochemical markers will be discussed in more detail in the next section.

**Figure 2:**
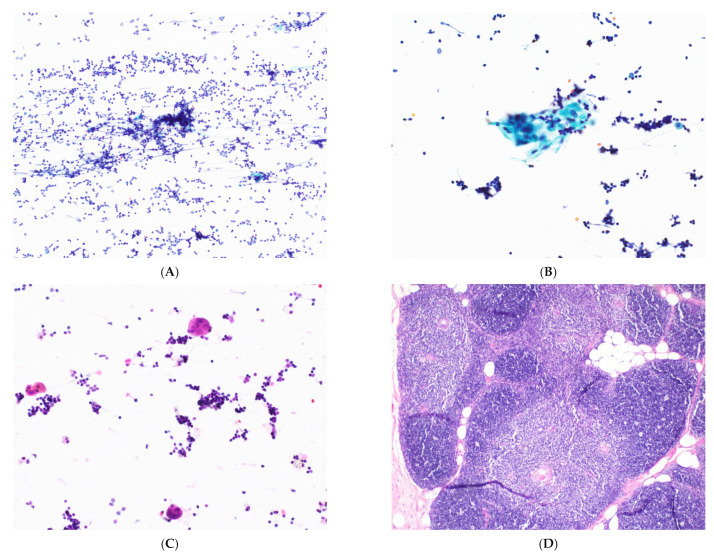
Thymic hyperplasia. (**A**) Low-power view of a thymic smear with small clusters of lymphoid and epithelial cells on a background of lymphocytes. H&E stain, 10× objective. The epithelial cell clusters can mimic lymphoid tangles in lymph node aspirates. They are typically smaller and sparser than those seen in thymoma. (**B**,**C**) The squamoid cells of Hassall’s corpuscles are rarely seen in aspirates, but when present they favor a diagnosis of thymic hyperplasia over thymoma. Pap and H&E stains, 40× objectives. (**D**) Resection specimen showing thymic hyperplasia, characterized by lymphoid follicles, Hassall’s corpuscles, and retained lobular thymic architecture. H&E stain, 10× objective.

The cytological diagnosis of thymomas is highly challenging. The literature regarding the cytological features of thymoma is limited due to the rarity of the tumor, and most published reports come from case reports of ectopic thymomas [10,12,22]. The hallmark of thymoma cytology is the presence of two cell types: lymphocytes and epithelial cells. However, this feature also elicits a large differential diagnosis that includes benign thymic lesions and a mediastinal lymph node with metastatic carcinoma. The epithelial cell clusters in thymic hyperplasia are typically smaller and sparser than those seen in thymoma, and the presence of Hassall’s corpuscles (Figure 2) helps to suggest a diagnosis of thymic hyperplasia over thymoma. However, Hassall’s corpuscles are not easily identified on smears and the size and density of epithelial clusters may depend on the overall cellularity of the smear [23]. Therefore, the diagnosis of thymic hyperplasia in cytology is extremely difficult. Additionally, the presence of Hassall’s corpuscles does not exclude the presence of thymoma, as they can be seen in B1 thymomas.

The lymphocytes in normal thymic tissue, thymic follicular hyperplasia, and type B1 thymoma are indistinguishable by cytomorphology and immunophenotype. If abundant thymic epithelial cell clusters are detected on smears or via immunocytochemistry, a diagnosis of thymoma may be favored, if supported by radiographic findings. Though radiographic studies can often distinguish a thymic mass from thymic hyperplasia, up to 23% of cases of thymic hyperplasia mimic thymoma on chest CT scans due to significant distortion of the gland [24]. It has been suggested that magnetic resonance imaging (MRI) is the best diagnostic test to separate thymoma from thymic hyperplasia [25]. A clinical finding of myasthenia gravis is also not helpful as it can be seen in either condition [26].

**Figure 3:**
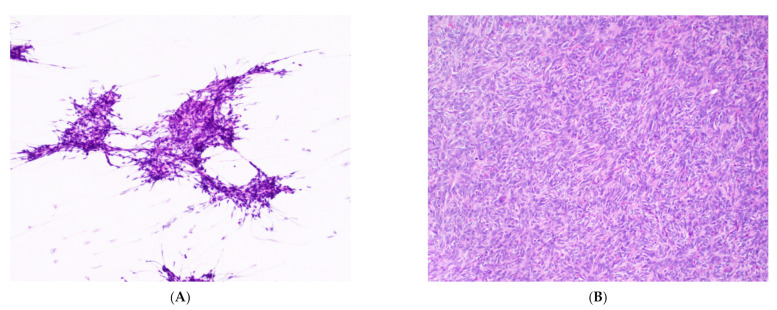
Type A thymoma. (**A**) Smear shows clusters of spindle-shaped epithelial cells on a background of few lymphocytes. H&E stain, 20× objective. (**B**) Corresponding spindle cells are seen in the surgical resection of type A thymoma. H&E stain, 20× objective.

**Figure 4:**
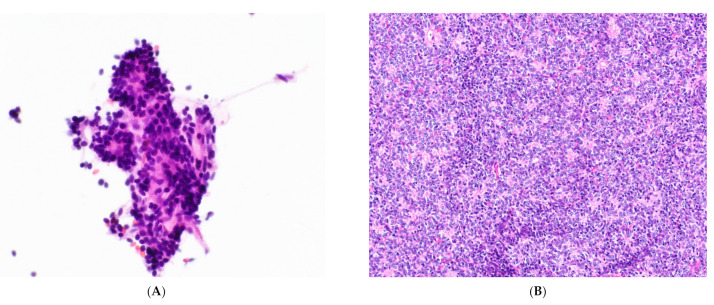
Type A thymoma with pseudo-rosettes mimicking neuroendocrine features. (**A**) Smear shows neuroendocrine-like rosettes. H&E stain, 60× objective. (**B**) Corresponding rosette formation is seen in the surgical resection of type A thymoma. H&E stain, 20× objective.

Aspirates of thymomas range from moderate to high cellularity, regardless of type [23]. Type A consists of clustered or loose spindle to ovoid epithelial cells on a clear or bloody background with few lymphocytes (Figure 3). The clustered epithelial cells may be observed forming a characteristic whirling arrangement or even pseudo-rosettes (Figure 4), as Type A thymomas can display a variety of histological patterns. The chromatin is finely dispersed with indistinct nucleoli. Naked nuclei, differentiated from lymphocytes by the complete absence of cytoplasm, may be seen but are uncommon. Crush artifact is rare in type A thymomas but may be seen in type B thymomas and within the type B epithelial component of type AB thymomas [27]. The presence of a spindle cell component along with moderate to large amounts of lymphocytes raises the possibility of a type AB thymoma.

**Figure 5:**
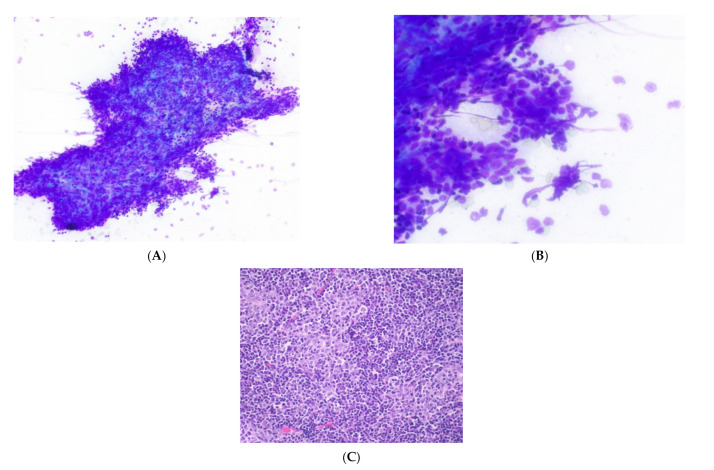
Type B thymoma. (**A**) Low-power and (**B**) high-power views of clustered round to ovoid epithelial cells of type B thymoma in close association with lymphocytes. Romanowsky stain, 20× & 60× objectives. (**C**) Surgical resection of type B2 thymoma showing clustered round epithelial cells in association with lymphocytes.

Aspirates of type B1 and B2 thymomas are predominately composed of lymphocytes with small aggregates of epithelial cells (Figure 5), while type B3 aspirates are composed of sheets, clusters, and loose epithelial cells on a background of few lymphocytes. The epithelial cells are bland with round to ovoid nuclei, smooth or slightly irregular nuclear contours, and finely granular chromatin. Type B2 and B3 thymomas may show enlarged cells and more prominent nucleoli [22,28]. The distinguishing features of thymic epithelial cells are better visualized on Papanicolaou-stained smears than on Romanowsky-stained smears [22]. The features of metaplastic thymoma and micronodular thymoma with lymphoid hyperplasia in cytological preparations have not been described.

**Figure 6:**
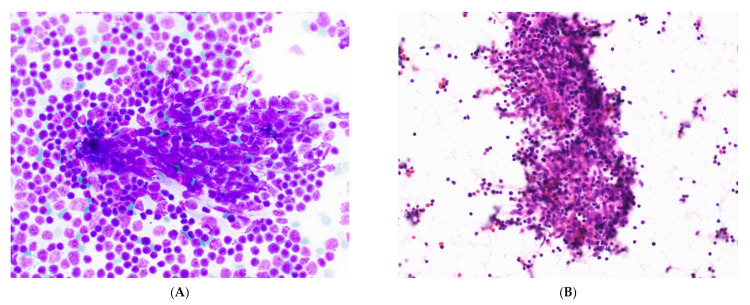
Type B thymoma mimicking a lymphoid lesion. (**A**) Epithelial cell clusters may be obscured by streaking crushed lymphocytes mimicking a lymphoid tangle. Romanowsky stain, 60× objective. (**B**) Epithelial cells may be detected in other regions of the smear but can still be challenging to type. H&E stain, 40× objective.

Another pitfall in the cytological diagnosis of thymoma is that aspirates can easily be miscategorized. The epithelial cells clusters in type B1 and B2 thymomas can be overshadowed by the benign lymphocytic background and be interpreted as lymphoid tangles, which may also raise concern for a low-grade lymphoma (Figure 6). In cases where lymphoma is suspected, flow cytometry may be suggested by a rapid on-site evaluator. Staining with pan-cytokeratin to highlight the epithelial cells is useful in these situations [22,29,30]. In thicker smears, thymoma epithelial cells may be confused with carcinomas. High-power examination of Pap- or H&E-stained smears should show that the epithelial cells of thymoma generally have open, smooth to finely granular chromatin in contrast to lymphoma and carcinoma [1,22]. Nonetheless, retrospective studies of thymic aspirates have shown that, through the use of cell blocks, ancillary tests (flow cytometry and immunocytochemical stains), and radiological findings, an experienced pathologist can provide accurate, clinically actionable diagnoses with thymic FNAs (accuracy 77–100%) [10,11,30,31].

Despite the aforementioned cytological characteristics, FNA is not a reliable means for classifying thymomas. Previous attempts to utilize epithelial cell morphology and lymphocyte-to-epithelial cell ratios have not shown significant correlations with surgical typing [22,27,32]. The clinical management of thymomas is not dependent on histological type but rather on clinical stage [10,30,33,34]. In addition, histological heterogeneity is common in any type of thymoma. Therefore, typing a thymoma in a cytology report is not advised. A diagnosis of “thymoma” provides adequate information to guide clinical management until staging information is available from the resection specimen.

## 3. Thymic Carcinoma

Thymic carcinomas constitute 14–22% of thymic epithelial neoplasms. They are usually asymptomatic and therefore frequently present at an advanced stage and carry an unfavorable prognosis [1,35]. Symptomatic cases are related to mass effect. Unlike thymomas, paraneoplastic syndromes are uncommon in thymic carcinomas. The WHO has classified thymic carcinomas by morphological subtype (Table 2) [1,36]. Histologically, thymic carcinomas are composed of malignant epithelial cells that are morphologically similar to carcinomas originating in other organs, usually with overt nuclear atypia and pleomorphism [1].

**Figure 7:**
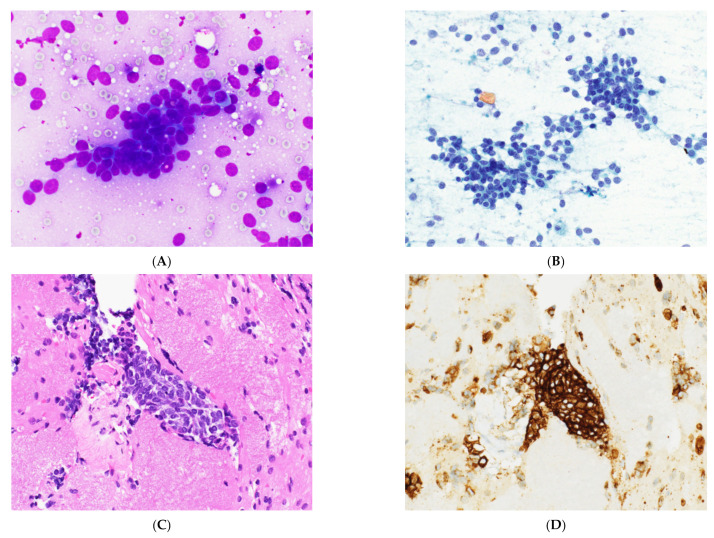
Thymic carcinoma. (**A**) Romanowsky and (**B**) Papanicalou stains of a thymic carcinoma aspirate show enlarged nucleoli with coarse chromatin. Neuroendocrine-like nuclear molding is seen. Magnification: 60× and 40× objectives. (**C**) The cell block shows positive staining for (**D**) CD117, supporting a diagnosis of thymic basaloid carcinoma. H&E and immunostain, 40×.

The cytological features in aspirates from thymic carcinoma vary by subtype, typically appearing, like their morphological analogs, elsewhere in the body. Squamous cell carcinoma, the most common subtype of thymic carcinoma, is also the most thoroughly described histological subtype in the cytology literature. This tumor shows epithelial cells that are overtly malignant, with enlarged nuclei, coarse chromatin, discrete nucleoli, and a moderate amount of cytoplasm (Figure 7) [1,22]. Thymic carcinoma smears frequently feature a necrotic background. However, necrosis can also be seen in thymoma aspirates, which may obscure non-malignant thymic epithelial cells, resulting in the misinterpretation of a thymoma as thymic carcinoma [10,37,38,39].

**Figure 8:**
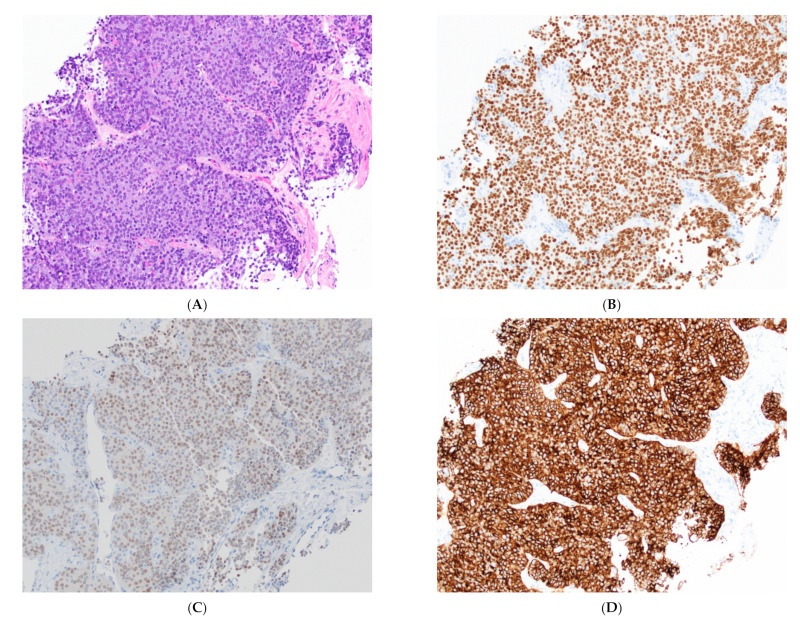
Immunocytochemical profile of thymic carcinoma. (**A**) H&E stain on a thymic carcinoma biopsy. (**B**) Staining with p63 highlights epithelial cells, and (**C**) reactivity for PAX8 favors thymic origin. (**D**) Reactivity for CD117 is highly specific for thymic carcinoma in this setting. The epithelial cells are negative for CD5 (not displayed), highlighting the poor sensitivity of immunocytochemical stains in thymic carcinoma. All images, 20× objective.

CD5 and CD117 (cKIT) are frequently positive in thymic carcinoma, with the sensitivity of each stain varying by subtype, but rarely positive in thymoma (Figure 8) [1]. Expression of MUC1, EZH2, and GLUT−1 (diffuse) favors thymic squamous cell carcinoma over type B3 thymoma [1,40,41,42,43]. Infiltrating lymphocytes in thymic carcinomas are mature T-cells and do not stain for CD99 or TdT, unlike the immature T-cells of thymomas [1].

Immunocytochemistry can also be used to distinguish carcinomas of thymic origin from metastasis from other sites. Both thymomas and thymic carcinomas are commonly positive for, in order of increasing sensitivity and specificity, FOXN1, polyclonal PAX8, and CD205, which may be useful in establishing the thymic origin of the tumor [18,19,44,45]. On the other hand, monoclonal PAX8 is typically negative in thymic epithelial neoplasms, though the results appear to vary by clone [46,47,48]. While PAX8 is commonly used in aspirates, the use of FOXN1 and CD205 has not been reported in cytology specimens.

Given the thoracic location, a primary lung neoplasm is high in the differential diagnosis. In cases of thymic squamous cell carcinoma, co-expression of CD5 and CD117 is highly specific, but only moderately sensitive, for thymic origin when compared to pulmonary squamous cell carcinomas [49]. There are scant data regarding the sensitivity and specificity of these two markers in other subtypes of thymic carcinoma. When PAX8 is included as evidence of thymic versus lung origin, reactivity for two of these three markers results in increased sensitivity for thymic origin relative to CD5 and CD117 alone [48].

The cytological features of many histological types of thymic carcinomas have not been thoroughly described in the literature. The cytological features of thymic carcinomas are summarized in Table 2.

## 4. Neuroendocrine Neoplasms

Thymic neuroendocrine neoplasms constitute 2–5% of thymic neoplasms. The WHO classification for thymic neuroendocrine neoplasms follows the classification of lung neuroendocrine neoplasms (Table 3) [1,50,51]. In order of increasing grade, they are histologically classified as typical carcinoid, atypical carcinoid, and neuroendocrine carcinoma (small and large cell types) based on mitotic count, the presence or absence of necrosis, and small versus large cell morphology [1]. Neuroendocrine neoplasms may show regions of different grades within the same lesion [52,53].

**Figure 9:**
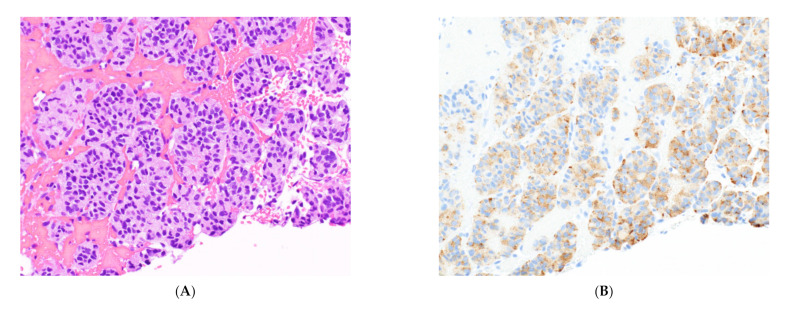

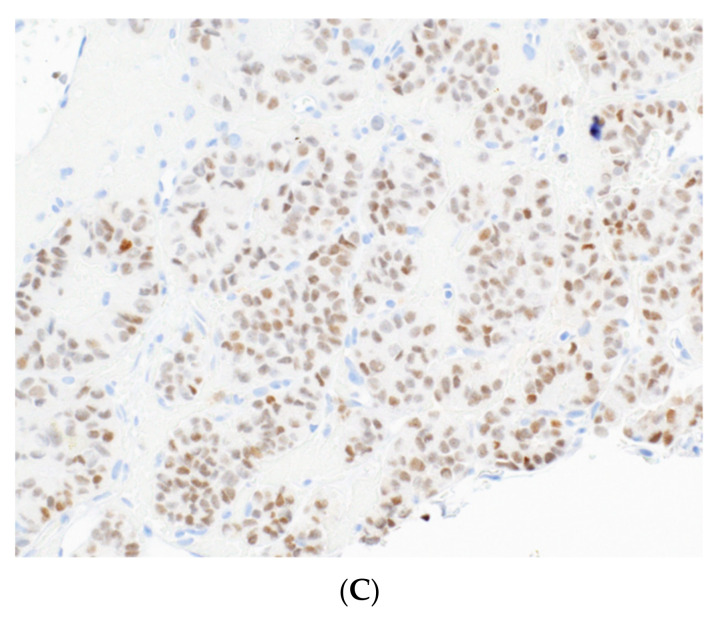
Thymic carcinoid tumor. (**A**) Small biopsy showing nests of cells with granular cytoplasm. H&E stain. Immunocytochemical stains of the same area show reactivity for (**B**) chromogranin, indicating neuroendocrine differentiation, and (**C**) PAX8, suggesting thymic origin. All images, 40× objective.

Aspirates of thymic carcinoid tumors show loose clusters or small strands of epithelial cells. The cells are uniform, small, and round to ovoid with scant cytoplasm and finely granular “salt and pepper” chromatin (Figure 9) [1]. Atypical nuclear features, including molding and hyperchromasia, are not observed. Necrosis and mitotic count, which are used to distinguish typical and atypical carcinoid tumors histologically, cannot be reliably used to distinguish these two entities on aspirates [1,22]. Carcinoid tumors can be easily misclassified due to their rarity and overlapping features with other entities. For example, pyknotic apoptotic nuclei can mimic lymphocytes, and the clustering of carcinoid cells resembles thymoma epithelial cells [54].

Immunocytochemistry for neuroendocrine markers, including chromogranin, synaptophysin, and INSM1, helps to classify tumor cells as neuroendocrine [55,56]. PAX8 can be expressed in thymic carcinoids [57,58]. The differential diagnosis of mediastinal neuroendocrine neoplasms also includes metastasis from other sites, such as medullary thyroid carcinoma (positive for calcitonin and monoclonal CEA), lung carcinoids (which may express TTF1), gastroenteropancreatic low- to intermediate-grade neuroendocrine tumors (which may express CDX2), and pancreatic neuroendocrine tumors (which may express PAX8 and progesterone receptor) [59,60,61,62,63,64]. Somatostatin-based imaging techniques may be required to resolve the site of origin [65]. Immunocytochemical staining may also be needed to rule out paraganglioma that can occur in the mediastinum. Paragangliomas are positive for GATA3, negative for cytokeratins, and S100 protein shows a sustentacular pattern [66,67,68].

Thymic neuroendocrine carcinomas, which include small cell carcinoma (SmCC) and large cell neuroendocrine carcinoma (LCNEC), are morphologically indistinguishable from lung SmCC and LCNEC. When presenting with advanced disease, it may also be clinically impossible to tell whether the tumor originated in the thymus or the lungs. While smoking is a known risk factor for pulmonary neuroendocrine carcinomas, there is currently no established link between any thymic neuroendocrine neoplasm and smoking, except in males with multiple endocrine neoplasia type 1 (associated with a higher risk of thymic carcinoids) [69].

SmCC aspirates show cells with a high nuclear-to-cytoplasmic ratio, scant cytoplasm, smudgy or finely granular chromatin, and nuclear molding on a background of necrosis and apoptosis. The “small cells” are approximately three times the size of lymphocytes. Crush artifact is common due to the fragility of the nuclei in SmCC. LCNEC aspirates show large ovoid to round cells, often on a background of necrosis. Prominent nucleoli and nuclear pleomorphism may be present such that adenocarcinoma enters the differential diagnosis. If neuroendocrine features are seen on cell block, positive immunocytochemistry for neuroendocrine markers may suggest the diagnosis. Although a definitive diagnosis of LCNEC versus SmCC is challenging, the presence of nucleoli and more abundant cytoplasm may favor LCNEC over SmCC [1,22].

## 5. Challenging Differential Diagnosis: Other Anterior Mediastinal Tumors and Ectopic Thymus

Based on the anterior mediastinal location, the differential diagnosis of thymic epithelial neoplasms includes mediastinal germ cell tumors, lymphomas, and metastatic tumors [2,70]. Mediastinal germ cell tumors can generally be distinguished from epithelial neoplasms based on clinical (serological), histological, and immunocytochemical findings, such as positivity for SALL4 and OCT3/4, and other more specific germ cell tumor markers [28,71]. The cytological features of embryonal carcinoma, yolk sac tumor, and choriocarcinoma are those of a high-grade carcinoma and they are rarely mistaken for features of a thymoma or a carcinoid tumor. Seminomas have a characteristic smearing pattern on cytology, tigroid background, and rarely enter the differential diagnosis of thymic epithelial tumors [72].

The most common anterior mediastinal lymphomas include Hodgkin lymphoma (most frequently the nodular sclerosing variant), diffuse large B-cell lymphoma, and lymphoblastic lymphoma [2,73]. Although rare, MALT lymphoma of the thymus may be seen, especially in patients with a history of Sjögren syndrome [10]. Low-grade thymic lymphomas are difficult to distinguish cytomorphologically from background thymic lymphocytes. Although epithelial cells are absent in lymphoma, sampling of the surrounding uninvolved thymic tissue can theoretically produce rare epithelial cells on smears, confounding the diagnosis [10]. Flow cytometry is useful in such instances, as the immature thymic lymphocytes present in thymic hyperplasia and thymoma can be distinguished from the clonal lymphocytes of lymphoproliferative disorders [74,75,76]. In cases where lymphoma is suspected, immunostaining and, when possible, flow cytometry should be used to verify the diagnosis.

**Figure 10:**
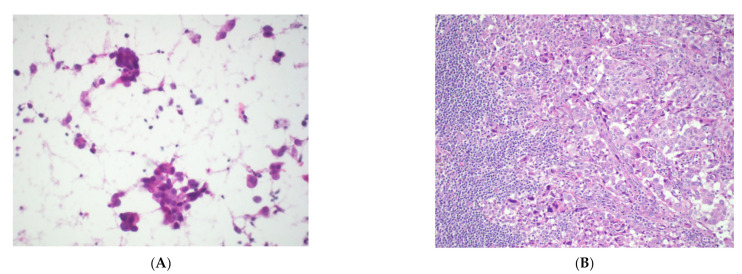

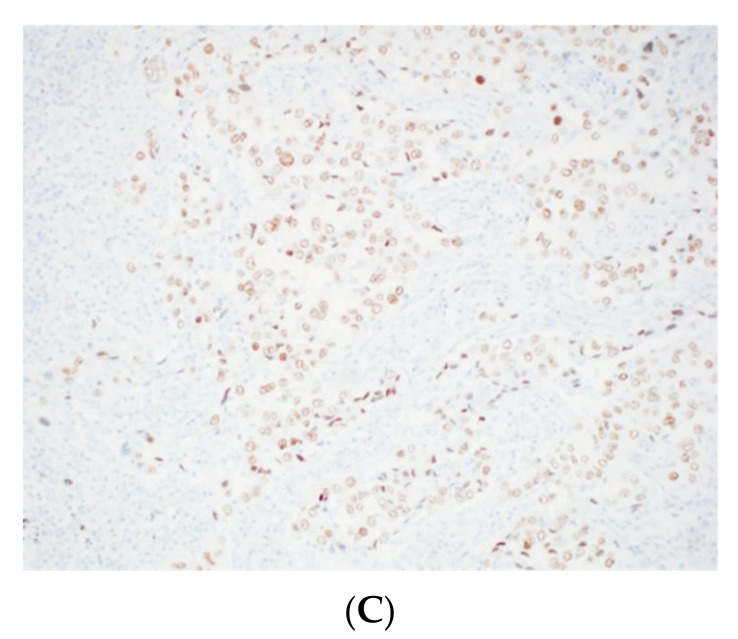
Metastatic lung adenocarcinoma. (**A**) Thymic smear showing overtly malignant cell clusters with mucinous cytoplasm suggestive of adenocarcinoma. H&E stain, 40× objective. (**B**) Resection shows an epithelial malignancy. Primary thymic and lung adenocarcinomas are morphologically indistinguishable. However, radiographic findings of a lung mass increase suspicion for a metastatic tumor. H&E stain, 20× objective. (**C**) Reactivity for TTF-1 rules out thymic origin and is consistent with a diagnosis of metastatic lung adenocarcinoma. Magnification: 20× objective.

Radiological information is often needed for an accurate diagnosis of mediastinal masses. In cases of thymic carcinoma, where cytological findings can be identical to those for carcinomas from other organs, a metastatic lesion should be radiologically excluded and ancillary tests should aid the diagnosis (Figure 10) [22,31,77]. While most thymic epithelial tumors occur in the anterior mediastinum, recurrent and metastatic tumors can present anywhere in the mediastinum, pleura, lungs, and other extrathoracic locations [78,79,80,81]. Additionally, ectopic thymomas have been reported in the lungs, middle mediastinum, thyroid, and other extramediastinal locations [10,82]. Due to their relative rarity, they may be misinterpreted if thymic neoplasms are not considered in the differential diagnosis.

## 6. Conclusions

Most thymomas are surgically excised without the need for preoperative diagnosis. Aspiration biopsy of thymic lesions, although uncommon, allows for the accurate categorization of a thymic mass as a primary thymic epithelial lesion versus a metastatic, germ cell, or hematopoietic neoplasm. This information is essential to guide clinical management.

Due to histological heterogeneity in thymomas, subtyping thymomas as WHO type A or B is not recommended in small biopsies. The pathologist should, however, indicate which component is present in the aspirate. The same should apply for the diagnosis of germ cell tumors, as not all components of a mixed germ cell tumor may be present in the aspirate.

## Figures and Tables

**Table 1:** Cytological features of thymoma *.

WHO Classification	Cytological Features	Differential Diagnosis
Type A (including atypical subtype)	Clusters of spindle or oval-shaped epithelial cells Scant lymphocytes	Mesenchymal neoplasm (including synovial sarcoma, myofibroblastic tumor, and follicular dendritic cell tumor) Sarcomatoid (spindle cell) carcinoma Sarcomatoid (spindle cell) mesothelioma Carcinoid tumor with spindle cell morphology Spindle cell melanoma
Type AB	Clusters of spindle or oval-shaped epithelial cells Abundant lymphocytes	Metastatic sarcomatoid carcinoma to lymph node
Type B1	Rare clusters of epithelial cells Abundant lymphocytes	Lymphoma Thymic hyperplasia Lymph node
Types B2 & B3	Clusters of epithelial cells Prominent nucleoli can be present Variable amounts of lymphocytes	Metastatic carcinoma to a lymph node
Metaplastic	Not described	Not described
Micronodular	Not described	Not described

* The lymphocytes in thymoma and thymic hyperplasia are immature T cells: CD3, CD5, TdT, and CD99 are positive by immunocytochemical staining and detectable by flow cytometry.

**Table 2:** Cytological features of thymic carcinoma *.

WHO Classification	Cytological Features
Squamous cell carcinoma	Overtly malignant features Hyperchromatic nuclei Prominent nucleoli Keratinization may or may not be present
Basaloid carcinoma	Cohesive clusters Monomorphic cells High nuclear-to-cytoplasmic ratio
Lymphoepithelial carcinoma	Overtly malignant features Prominent nucleoli Large vesicular nuclei Lymphocytes in the background Positive for EBV (chromogenic ISH stain available)
NUT carcinoma	Discohesive clusters Monomorphic cells Discrete to prominent nucleoli Positive for NUT (IHC stain available)
Clear cell carcinoma	Cohesive clusters Vacuolated cells (Scant reports)
Low-grade papillary adenocarcinoma	Not described
Mucoepidermoid carcinoma	Cohesive clusters Small-sized cells Mucinous background
Thymic carcinoma with adenoid cystic carcinoma-like features	Not described
Enteric-type adenocarcinoma	Clusters of malignant cells Nuclear crowding or picket-fence formation Positive for CDX2 (IHC)
Adenocarcinoma NOS	Not described
Adenosquamous carcinoma	Not described
Sarcomatoid carcinoma	Not described
Undifferentiated carcinoma	Not described
Thymic carcinoma NOS	Not described

* Positive immunocytochemical staining for CD5, CD117, and FOXN1 in malignant epithelial cells is helpful to suggest thymic origin. The lymphocytes in thymic carcinomas are mature and therefore negative for TdT. The epithelial cells of thymomas are usually negative for CD5 and CD117 and are only rarely positive.

**Table 3:** Cytological features of thymic neuroendocrine neoplasms.

WHO Classification	Cytological Features
Carcinoid tumor	Uniform round to ovoid cells Finely granular cytoplasm “Salt and pepper” chromatin
Small cell carcinoma	High nuclear-to-cytoplasmic ratio Nuclear molding Inconspicuous or absent nucleoli Fine chromatin pattern +/− Necrosis
Large cell neuroendocrine carcinoma	High nuclear to cytoplasmic ratio Nuclear molding Prominent nucleoli Fine chromatin pattern +/− Necrosis
